# High-resolution computed tomography reconstructions of invertebrate burrow systems

**DOI:** 10.1038/sdata.2015.52

**Published:** 2015-09-21

**Authors:** Rachel Hale, Richard Boardman, Mark N. Mavrogordato, Ian Sinclair, Trevor J. Tolhurst, Martin Solan

**Affiliations:** 1 Ocean and Earth Science, National Oceanography Centre, Southampton, University of Southampton, Waterfront Campus, European Way, Southampton SO14 3ZH, UK; 2 University of Southampton, Engineering and the Environment, Highfield, Southampton SO17 1BJ, UK; 3 University of East Anglia, School of Environmental Science, Norwich Research Park, Norwich NR4 7TJ, UK

**Keywords:** Animal behaviour, Ecosystem ecology, 3-D reconstruction, Biooceanography

## Abstract

The architecture of biogenic structures can be highly influential in determining species contributions to major soil and sediment processes, but detailed 3-D characterisations are rare and descriptors of form and complexity are lacking. Here we provide replicate high-resolution micro-focus computed tomography (μ-CT) data for the complete burrow systems of three co-occurring, but functionally contrasting, sediment-dwelling inter-tidal invertebrates assembled alone, and in combination, in representative model aquaria. These data (≤2,000 raw image slices aquarium^−1^, isotropic voxel resolution, 81 μm) provide reference models that can be used for the development of novel structural analysis routines that will be of value within the fields of ecology, pedology, geomorphology, palaeobiology, ichnology and mechanical engineering. We also envisage opportunity for those investigating transport networks, vascular systems, plant rooting systems, neuron connectivity patterns, or those developing image analysis or statistics related to pattern or shape recognition. The dataset will allow investigators to develop or test novel methodology and ideas without the need to generate a complete three-dimensional computation of exemplar architecture.

## Background & Summary

Soils and sediments provide habitat for a wide range of organisms and the vertical exploitation of this ecospace has been important in mediating major ecosystem properties and the diversification of life over geological timescales^1–3^. Insights about organism-sediment relations, however, have largely been restricted to two dimensions^4,5^, although important inferences about burrowing mechanics^6^ and three dimensional architecture^7^ have been made from burrow castings^8^ and the use of optically transparent sediment analogues^9^. Relatively few studies apply non-invasive interrogation of intact sedimentary media^10–13^, despite significant advances in optical and clinical imaging technology^14^. High-resolution micro-focus computed tomography (μ-CT) offers a way of not only imaging the organisms themselves^15,16^ but also visualising the structure of a whole sediment core in three dimensions to allow quantitative examination of organismal burrowing^17^.

Experimental details are given in Hale *et al.*^18^. Briefly, surficial sediment (less than 3 cm depth; mean particle size, 54.80 μm; mud content, 55.93%) and three co-occurring functionally contrasting inter-tidal invertebrates (the polychaete *Hediste diversicolor*, the gastropod *Hydrobia ulvae* and mud shrimp *Corophium volutator*) were collected from the mid-shore at Breydon water, Great Yarmouth, UK (N52° 37.030′, E01° 41.390′) and returned to the *Biodiversity and Ecosystem Futures Facility* at the University of Southampton to acclimatise to laboratory conditions (5 days). Sediment was sieved (500 μm mesh) in a seawater (sand filtered, UV sterilized and salinity 33 practical salinity units) bath to remove macrofauna and allowed to settle for 48 h to retain the fine fraction (less than 63 μm). Circular aquaria (internal diameter=10 cm, 15 cm tall, n=20) were filled to a depth of 8 cm with sediment homogenate overlain by 4 cm of seawater.

Overlying seawater was replaced after 24 h to remove excess nutrients associated with assembly. Aquaria were aerated and maintained at 12±0.1 °C under a 12:12 h light (Aqualine T5 Reef White 10 K fluorescent light tubes, Aqua Medic) cycle. Fauna were not added until the lower regions of the sediment cores showed evidence of reducing conditions (visible anoxic microniche formation). Replicate (*n*=5) invertebrate communities (1 g wet weight aquaria^−1^; ~127 g m^−2^) were assembled in monoculture (*Hediste diversicolor*, HD; *Hydrobia ulvae*, HU; or *Corophium volutator*, CV) and in equal mixture (Mix).

These μ-CT sediment scans can provide reference models which may be of use in a range of connected fields, such as for the development of novel structural analysis routines and computer models in ecology^17,19^, pedology^20^, geomorphology, ichnology^21^, palaeobiology, and mechanical engineering^22^. We envisage those investigating transport networks^23^, vascular systems, plant rooting systems^24^, neuron connectivity patterns^25,26^, or developing image analysis or statistics related to pattern or shape recognition will find these data of interest. We have made this dataset available to allow investigators to develop or test novel methodology and ideas without the need to generate a complete three-dimensional computation of exemplar architecture.

## Methods

**Figure 1:**
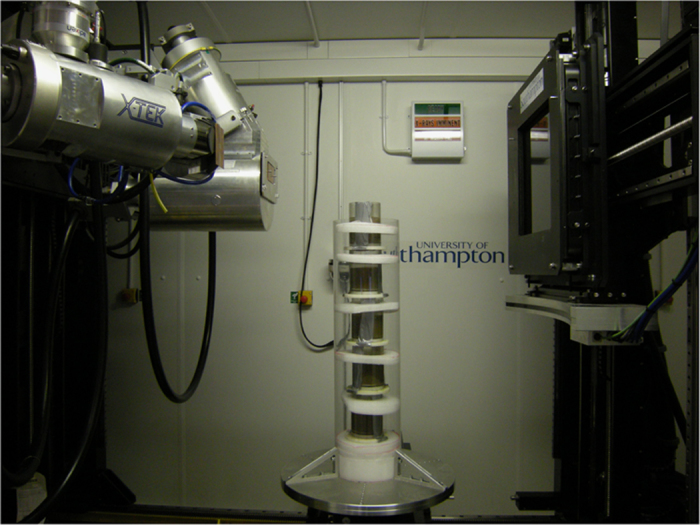
Five aquaria stacked in the holding brace in the micro-focus computed tomography scanner housed within the μ-VIS X-ray Imaging Centre, University of Southampton.

Reconstruction of biogenic structures in the aquaria was achieved using a 225/450 kVp Nikon/Metris custom designed micro-focus computed tomography scanner housed within the μ-VIS X-ray Imaging Centre, University of Southampton. As the system used to acquire the scan data requires the cores to be held vertically batches of 5 aquaria were stacked and secured in a custom-made holding brace to ensure stability and prevent sediment or seawater leakage during rotation and scanning (Fig. 1). During each acquisition, the aquaria were rotated through 360° whilst collecting 3,142 projections averaging over 8 frames per 250 ms projection (for a total of 2 s per projection, ca. 105 min per acquisition). Ring artifact reduction was enabled. X-ray conditions were set to 300 kVp and 326 μA with a 3 mm Cu filter, and an XRD 1621 CN3 H5 PerkinElmer flat panel detector (CsI scintillator) was used to collect the images. In the resulting reconstructed images, levels of grey scale reflect the level of X-ray attenuation caused by variation in bulk density^3^. Hence, brighter pixels represent denser material (sediment) and darker pixels represent less dense material (burrow voids). Raw image slices (*n*=2,000 aquarium^−1^, voxel resolution=81 μm) were processed as follows: First, the projection data was reconstructed using *CTPro3D* (v. XT 2.2 service pack 10, Nikon Metrology, UK) and *CTAgent* (v. XT 2.2 service pack 10, Nikon Metrology, UK). The reconstructed volumes were converted to 8 bit format using FIJI^27^ (Version 1.49a) to reduce file sizes and computational loading. Finally, these images were opened as a 3D project in *VGStudio Max* (v. 2.1 Volume Graphics GmbH, Germany) and an edge-preserving 3D 5 pixel non-linear digital median filter was applied to reduce noise in the images.

**Figure 2:**
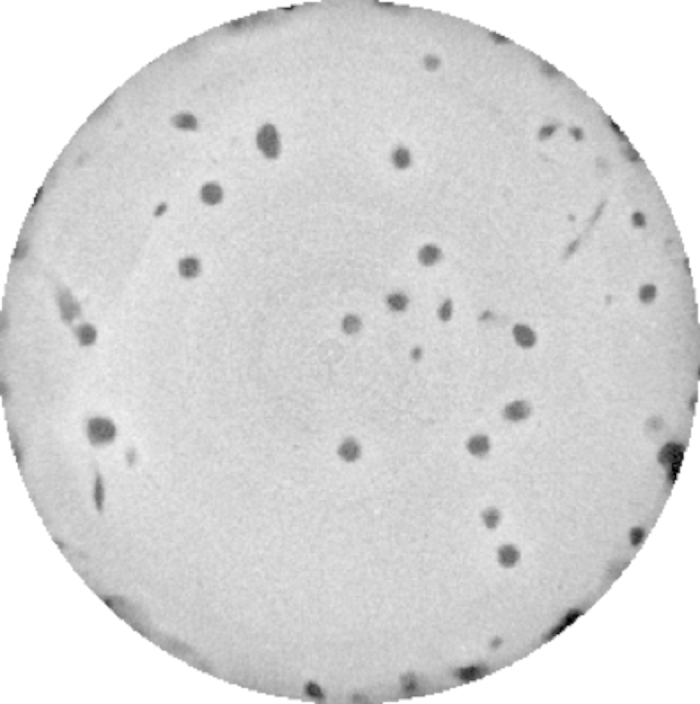
A representative transverse core slice from the Core Volumes image set showing distinct low density burrows (dark grey) through the (light grey) higher density sediment. A Core Volume image set consists of number images that are sequentially stacked to create the three-dimensional core volume image. The central sediment core is 10 cm in diameter.

**Figure 3:**
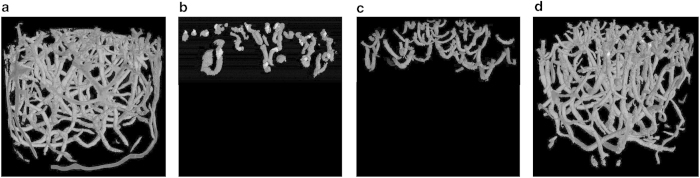
Representative example reconstructed three-dimensional burrow models for (**a**) *Hediste diversicolor*, (**b**) *Hydrobia ulvae*, (**c**) *Corophium volutator*, and (**d**) the three species in mixture created from the stacked Burrow Volumes images in VG Studio. The sediment cores containing the burrows are 10 cm in diameter.

Three types of images were produced. Whole core scans of 16-bit quality (Core_Volume_01_16bit to Core_Volume_20_16bit: Data Citation 1), processed image whole core scans of 8-bit quality with a 3D 5 pixel non-linear digital median filter applied (Core_Volume_01 to Core_Volume_20: Data Citation 1), an example slice of which is shown in Fig. 2, and processed burrow images (Burrow_Volume_01 to Burrow_Volume_20: Data Citation 1). To produce the burrow images the three-dimensional image captured of the aquaria and the holding brace was discarded to leave the central sediment core volume. Within the sediment core, regions of interest, the low density burrows, were segmented using a threshold based seed point growing algorithm that identified three-dimensional areas of similar low densities to produce a three-dimensional image of the burrow network (Fig. 3) called the burrow volume.

## Data Records

All data records listed in this section are available at the Harvard Dataverse (Data Citation 1). Details of supplementary experimental procedures and additional materials, including videos of the three dimensional burrow structures are available from Hale *et al.*^18^. Computed tomography three-dimensional files have been converted to stacked tagged image file format (TIFF) images with associated dimension data (image width, image breadth, stack height) to enable access by multiple processing programs. There are three sets of images (*n*=20). Sediment core volume images for each replicate in 16- bit (Core_Volume_01_16bit to Core_Volume_20_16bit) and 8- bit (Core_Volume_01 to Core_Volume_20) and burrow volume images for each replicate (Burrow_Volume_01 to Burrow_Volume_20).

## Technical Validation

The system geometry at the μ-VIS X-ray Imaging Centre, University of Southampton, is checked and validated periodically using a 3 ruby sphere reference object that has been measured using optical profilometry (Xyris 4000 CL Surface Profiler, Taicaan technologies Europe). The centroid distances (threshold independent) of these ruby spheres when measured using CT are in agreement with the optical profilometry measurements to within 0.2%. For the presented scans, measurement validation was carried out post-scan by ensuring reference distances were accurately represented in the final images (within 1%).

## Usage Notes

The TIFF images provided should be imported as a three dimensional image sequence. The starting image is 0. The number of images and dimensions of each stack for each sediment core or burrow volume is provided in Tables 1,2,3 (available online only). When importing, image names should be sorted numerically.

There are no limitations on data use.

## Additional Information

Tables 1,2,3 are only available in the online version of this paper.

**How to cite this article:** Hale, R. *et al.* High-resolution computed tomography reconstructions of invertebrate burrow systems. *Sci. Data* 2:150052 doi: 10.1038/sdata.2015.52 (2015).

## Supplementary Material



## Figures and Tables

**Table 1:** Stacked image data for the 16-bit quality unfiltered sediment core volumes (*n*=20)

**Core_ID**	**Scan_ID**	**Replicate_ID**	**Species_ID**	**dimension_X**	**dimension_y**	**dimension_Z**	**number_of_images**	**species_id_folder**	**stack_folder**	**imagestack_fileprefix**	**first_image**	**last_image**
34	1	Rep_01	Corophium volutator	2000	2000	2000	2000	Species_Corophium	Core_Volume_01_CV01_16bit	Core_Volume_01_CV01_16bit_2000_2000_2000_	Core_Volume_01_CV01_16bit_2000_2000_2000_0000.tif	Core_Volume_01_CV01_16bit_2000_2000_2000_1999.tif
24	2	Rep_01	Hediste diversicolor	2000	2000	2000	2000	Species_Hediste	Core_Volume_02_HD01_16bit	Core_Volume_02_HD01_16bit_2000_2000_2000_	Core_Volume_02_HD01_16bit_2000_2000_2000_0000.tif	Core_Volume_02_HD01_16bit_2000_2000_2000_1999.tif
39	3	Rep_01	Mixed species	2000	2000	2000	2000	Species_Mixed	Core_Volume_03_Mx01_16bit	Core_Volume_03_Mx01_16bit_2000_2000_2000_	Core_Volume_03_Mx01_16bit_2000_2000_2000_0000.tif	Core_Volume_03_Mx01_16bit_2000_2000_2000_1999.tif
29	4	Rep_01	Hydrobia ulvae	2000	2000	2000	2000	Species_Hydrobia	Core_Volume_04_HU01_16bit	Core_Volume_04_HU01_16bit_2000_2000_2000_	Core_Volume_04_HU01_16bit_2000_2000_2000_0000.tif	Core_Volume_04_HU01_16bit_2000_2000_2000_1999.tif
40	5	Rep_02	Mixed species	2000	2000	2000	2000	Species_Mixed	Core_Volume_05_Mx02_16bit	Core_Volume_05_Mx02_16bit_2000_2000_2000_	Core_Volume_05_Mx02_16bit_2000_2000_2000_0000.tif	Core_Volume_05_Mx02_16bit_2000_2000_2000_1999.tif
23	6	Rep_02	Hediste diversicolor	2000	2000	2000	2000	Species_Hediste	Core_Volume_06_HD02_16bit	Core_Volume_06_HD02_16bit_2000_2000_2000_	Core_Volume_06_HD02_16bit_2000_2000_2000_0000.tif	Core_Volume_06_HD02_16bit_2000_2000_2000_1999.tif
36	7	Rep_03	Mixed species	2000	2000	2000	2000	Species_Mixed	Core_Volume_07_Mx03_16bit	Core_Volume_07_Mx03_16bit_2000_2000_2000_	Core_Volume_07_Mx03_16bit_2000_2000_2000_0000.tif	Core_Volume_07_Mx03_16bit_2000_2000_2000_1999.tif
31	8	Rep_02	Corophium volutator	2000	2000	2000	2000	Species_Corophium	Core_Volume_08_CV02_16bit	Core_Volume_08_CV02_16bit_2000_2000_2000_	Core_Volume_08_CV02_16bit_2000_2000_2000_0000.tif	Core_Volume_08_CV02_16bit_2000_2000_2000_1999.tif
30	9	Rep_02	Hydrobia ulvae	2000	2000	2000	2000	Species_Hydrobia	Core_Volume_09_HU02_16bit	Core_Volume_09_HU02_16bit_2000_2000_2000_	Core_Volume_09_HU02_16bit_2000_2000_2000_0000.tif	Core_Volume_09_HU02_16bit_2000_2000_2000_1999.tif
25	10	Rep_03	Hediste diversicolor	2000	2000	2000	2000	Species_Hediste	Core_Volume_10_HD03_16bit	Core_Volume_10_HD03_16bit_2000_2000_2000_	Core_Volume_10_HD03_16bit_2000_2000_2000_0000.tif	Core_Volume_10_HD03_16bit_2000_2000_2000_1999.tif
28	11	Rep_03	Hydrobia ulvae	2000	2000	2000	2000	Species_Hydrobia	Core_Volume_11_HU03_16bit	Core_Volume_11_HU03_16bit_2000_2000_2000_	Core_Volume_11_HU03_16bit_2000_2000_2000_0000.tif	Core_Volume_11_HU03_16bit_2000_2000_2000_1999.tif
38	12	Rep_04	Mixed species	2000	2000	2000	2000	Species_Mixed	Core_Volume_12_Mx04_16bit	Core_Volume_12_Mx04_16bit_2000_2000_2000_	Core_Volume_12_Mx04_16bit_2000_2000_2000_0000.tif	Core_Volume_12_Mx04_16bit_2000_2000_2000_1999.tif
22	13	Rep_04	Hediste diversicolor	2000	2000	2000	2000	Species_Hediste	Core_Volume_13_HD04_16bit	Core_Volume_13_HD04_16bit_2000_2000_2000_	Core_Volume_13_HD04_16bit_2000_2000_2000_0000.tif	Core_Volume_13_HD04_16bit_2000_2000_2000_1999.tif
27	14	Rep_04	Hydrobia ulvae	2000	2000	2000	2000	Species_Hydrobia	Core_Volume_14_HU04_16bit	Core_Volume_14_HU04_16bit_2000_2000_2000_	Core_Volume_14_HU04_16bit_2000_2000_2000_0000.tif	Core_Volume_14_HU04_16bit_2000_2000_2000_1999.tif
35	15	Rep_03	Corophium volutator	2000	2000	2000	2000	Species_Corophium	Core_Volume_15_CV03_16bit	Core_Volume_15_CV03_16bit_2000_2000_2000_	Core_Volume_15_CV03_16bit_2000_2000_2000_0000.tif	Core_Volume_15_CV03_16bit_2000_2000_2000_1999.tif
32	16	Rep_04	Corophium volutator	2000	2000	2000	2000	Species_Corophium	Core_Volume_16_CV04_16bit	Core_Volume_16_CV04_16bit_2000_2000_2000_	Core_Volume_16_CV04_16bit_2000_2000_2000_0000.tif	Core_Volume_16_CV04_16bit_2000_2000_2000_1999.tif
37	17	Rep_05	Mixed species	2000	2000	2000	2000	Species_Mixed	Core_Volume_17_Mx05_16bit	Core_Volume_17_Mx05_16bit_2000_2000_2000_	Core_Volume_17_Mx05_16bit_2000_2000_2000_0000.tif	Core_Volume_17_Mx05_16bit_2000_2000_2000_1999.tif
33	18	Rep_05	Corophium volutator	2000	2000	2000	2000	Species_Corophium	Core_Volume_18_CV05_16bit	Core_Volume_18_CV05_16bit_2000_2000_2000_	Core_Volume_18_CV05_16bit_2000_2000_2000_0000.tif	Core_Volume_18_CV05_16bit_2000_2000_2000_1999.tif
26	19	Rep_05	Hydrobia ulvae	2000	2000	2000	2000	Species_Hydrobia	Core_Volume_19_HU05_16bit	Core_Volume_19_HU05_16bit_2000_2000_2000_	Core_Volume_19_HU05_16bit_2000_2000_2000_0000.tif	Core_Volume_19_HU05_16bit_2000_2000_2000_1999.tif
21	20	Rep_05	Hediste diversicolor	2000	2000	2000	2000	Species_Hediste	Core_Volume_20_HD05_16bit	Core_Volume_20_HD05_16bit_2000_2000_2000_	Core_Volume_20_HD05_16bit_2000_2000_2000_0000.tif	Core_Volume_20_HD05_16bit_2000_2000_2000_1999.tif

**Table 2:** Stacked image data for the 8-bit quality sediment core volumes with 3D 5 pixel non-linear digital median filtered applied (*n*=20)

**Core_ID**	**Scan_ID**	**Replicate_ID**	**Species_ID**	**dimension_X**	**dimension_y**	**dimension_Z**	**number_of_images**	**species_id_folder**	**stack_folder**	**imagestack_fileprefix**	**first_image**	**last_image**
34	1	Rep_01	Corophium volutator	1230	1230	700	700	Species_Corophium	Core_Volume_01_CV01	Core_Volume_01_CV01_1230_1230_700_	Core_Volume_01_CV01_1230_1230_700_0000.tif	Core_Volume_01_CV01_1230_1230_700_0699.tif
24	2	Rep_01	Hediste diversicolor	1701	1401	928	928	Species_Hediste	Core_Volume_02_HD01	Core_Volume_02_HD01_1701_1401_928_	Core_Volume_02_HD01_1701_1401_928_0000.tif	Core_Volume_02_HD01_1701_1401_928_0927.tif
39	3	Rep_01	Mixed species	1401	1401	892	892	Species_Mixed	Core_Volume_03_Mx01	Core_Volume_03_Mx01_1401_1401_892_	Core_Volume_03_Mx01_1401_1401_892_0000.tif	Core_Volume_03_Mx01_1401_1401_829_0828.tif
29	4	Rep_01	Hydrobia ulvae	1501	1501	1201	1201	Species_Hydrobia	Core_Volume_04_HU01	Core_Volume_04_HU01_1501_1501_1201_	Core_Volume_04_HU01_1501_1501_1201_0000.tif	Core_Volume_04_HU01_1501_1501_1201_1200.tif
40	5	Rep_02	Mixed species	1207	1217	923	923	Species_Mixed	Core_Volume_05_Mx02	Core_Volume_05_Mx02_1207_1217_923_	Core_Volume_05_Mx02_1207_1217_923_0000.tif	Core_Volume_05_Mx02_1207_1217_923_0922.tif
23	6	Rep_02	Hediste diversicolor	1601	1601	1401	1401	Species_Hediste	Core_Volume_06_HD02	Core_Volume_06_HD02_1601_1601_1401_	Core_Volume_06_HD02_1601_1601_1401_0000.tif	Core_Volume_06_HD02_1601_1601_1401_1400.tif
36	7	Rep_03	Mixed species	1501	1501	1101	1101	Species_Mixed	Core_Volume_07_Mx03	Core_Volume_07_Mx03_1501_1501_1101_	Core_Volume_07_Mx03_1501_1501_1101_0000.tif	Core_Volume_07_Mx03_1501_1501_1101_1100.tif
31	8	Rep_02	Corophium volutator	1501	1501	1401	1401	Species_Corophium	Core_Volume_08_CV02	Core_Volume_08_CV02_1501_1501_1401_	Core_Volume_08_CV02_1501_1501_1401_0000.tif	Core_Volume_08_CV02_1501_1501_1401_1400.tif
30	9	Rep_02	Hydrobia ulvae	1501	1501	801	801	Species_Hydrobia	Core_Volume_09_HU02	Core_Volume_09_HU02_1501_1501_801_	Core_Volume_09_HU02_1501_1501_801_0000.tif	Core_Volume_09_HU02_1501_1501_801_0800.tif
25	10	Rep_03	Hediste diversicolor	2000	2000	2000	2000	Species_Hediste	Core_Volume_10_HD03	Core_Volume_10_HD03_2000_2000_2000_	Core_Volume_10_HD03_2000_2000_2000_0000.tif	Core_Volume_10_HD03_2000_2000_2000_1999.tif
28	11	Rep_03	Hydrobia ulvae	1501	1501	1001	1001	Species_Hydrobia	Core_Volume_11_HU03	Core_Volume_11_HU03_1501_1501_1001_	Core_Volume_11_HU03_1501_1501_1001_0000.tif	Core_Volume_11_HU03_1501_1501_1001_1000.tif
38	12	Rep_04	Mixed species	1601	1601	1301	1301	Species_Mixed	Core_Volume_12_Mx04	Core_Volume_12_Mx04_1601_1601_1301_	Core_Volume_12_Mx04_1601_1601_1301_0000.tif	Core_Volume_12_Mx04_1601_1601_1301_1300.tif
22	13	Rep_04	Hediste diversicolor	1601	1601	1301	1301	Species_Hediste	Core_Volume_13_HD04	Core_Volume_13_HD04_1601_1601_1301_	Core_Volume_13_HD04_1601_1601_1301_0000.tif	Core_Volume_13_HD04_1601_1601_1301_1300.tif
27	14	Rep_04	Hydrobia ulvae	1601	1601	951	951	Species_Hydrobia	Core_Volume_14_HU04	Core_Volume_14_HU04_1601_1601_951_	Core_Volume_14_HU04_1601_1601_951_0000.tif	Core_Volume_14_HU04_1601_1601_951_0950.tif
35	15	Rep_03	Corophium volutator	1601	1601	1201	1201	Species_Corophium	Core_Volume_15_CV03	Core_Volume_15_CV03_1601_1601_1201_	Core_Volume_15_CV03_1601_1601_1201_0000.tif	Core_Volume_15_CV03_1601_1601_1201_1200.tif
32	16	Rep_04	Corophium volutator	1601	1601	1001	1001	Species_Corophium	Core_Volume_16_CV04	Core_Volume_16_CV04_1601_1601_1001_	Core_Volume_16_CV04_1601_1601_1001_0000.tif	Core_Volume_16_CV04_1601_1601_1001_1000.tif
37	17	Rep_05	Mixed species	1601	1601	1501	1501	Species_Mixed	Core_Volume_17_Mx05	Core_Volume_17_Mx05_1601_1601_1501_	Core_Volume_17_Mx05_1601_1601_1501_0000.tif	Core_Volume_17_Mx05_1601_1601_1501_1500.tif
33	18	Rep_05	Corophium volutator	1601	1601	1201	1201	Species_Corophium	Core_Volume_18_CV05	Core_Volume_18_CV05_1601_1601_1201_	Core_Volume_18_CV05_1601_1601_1201_0000.tif	Core_Volume_18_CV05_1601_1601_1201_1200.tif
26	19	Rep_05	Hydrobia ulvae	1601	1601	1101	1101	Species_Hydrobia	Core_Volume_19_HU05	Core_Volume_19_HU05_1601_1601_1101_	Core_Volume_19_HU05_1601_1601_1101_0000.tif	Core_Volume_19_HU05_1601_1601_1101_1100.tif
21	20	Rep_05	Hediste diversicolor	1601	1601	1401	1401	Species_Hediste	Core_Volume_20_HD05	Core_Volume_20_HD05_1601_1601_1401_	Core_Volume_20_HD05_1601_1601_1401_0000.tif	Core_Volume_20_HD05_1601_1601_1401_1400.tif

**Table 3:** Stacked image data for the burrow volumes (*n*=20)

**Core_ID**	**Scan_ID**	**Replicate_ID**	**Species_ID**	**dimension_X**	**dimension_y**	**dimension_Z**	**number_of_images**	**species_id_folder**	**stack_folder**	**imagestack_fileprefix**	**first_image**	**last_image**
34	1	Rep_01	Corophium volutator	1229	1184	265	265	Species_Corophium	Burrow_Volume_01_CV01	Burrow_Volume_01_CV01_1229_1184_265_	Burrow_Volume_01_CV01_1229_1184_265_000.tif	Burrow_Volume_01_CV01_1229_1184_265_264.tif
24	2	Rep_01	Hediste diversicolor	1207	1219	909	909	Species_Hediste	Burrow_Volume_02_HD01	Burrow_Volume_02_HD01_1207_1219_909_	Burrow_Volume_02_HD01_1207_1219_909_000.tif	Burrow_Volume_02_HD01_1207_1219_909_908.tif
39	3	Rep_01	Mixed species	1219	1236	872	872	Species_Mixed	Burrow_Volume_03_Mx01	Burrow_Volume_03_Mx01_1219_1236_872_	Burrow_Volume_03_Mx01_1219_1236_872_000.tif	Burrow_Volume_03_Mx01_1219_1236_872_871.tif
29	4	Rep_01	Hydrobia ulvae	1220	1133	411	411	Species_Hydrobia	Burrow_Volume_04_HU01	Burrow_Volume_04_HU01_1220_1133_411_	Burrow_Volume_04_HU01_1220_1133_411_000.tif	Burrow_Volume_04_HU01_1220_1133_411_410.tif
40	5	Rep_02	Mixed species	1205	1215	900	900	Species_Mixed	Burrow_Volume_05_Mx02	Burrow_Volume_05_Mx02_1205_1215_900_	Burrow_Volume_05_Mx02_1205_1215_900_000.tif	Burrow_Volume_05_Mx02_1205_1215_900_899.tif
23	6	Rep_02	Hediste diversicolor	1228	1224	906	906	Species_Hediste	Burrow_Volume_06_HD02	Burrow_Volume_06_HD02_1228_1224_906_	Burrow_Volume_06_HD02_1228_1224_906_000.tif	Burrow_Volume_06_HD02_1228_1224_906_905.tif
36	7	Rep_03	Mixed species	1228	1223	898	898	Species_Mixed	Burrow_Volume_07_Mx03	Burrow_Volume_07_Mx03_1228_1223_898_	Burrow_Volume_07_Mx03_1228_1223_898_000.tif	Burrow_Volume_07_Mx03_1228_1223_898_897.tif
31	8	Rep_02	Corophium volutator	1123	1121	220	220	Species_Corophium	Burrow_Volume_08_CV02	Burrow_Volume_08_CV02_1123_1121_220_	Burrow_Volume_08_CV02_1123_1121_220_000.tif	Burrow_Volume_08_CV02_1123_1121_220_219.tif
30	9	Rep_02	Hydrobia ulvae	900	1212	313	313	Species_Hydrobia	Burrow_Volume_09_HU02	Burrow_Volume_09_HU02_900_1212_313_	Burrow_Volume_09_HU02_900_1212_313_000.tif	Burrow_Volume_09_HU02_900_1212_313_312.tif
25	10	Rep_03	Hediste diversicolor	1220	1235	854	854	Species_Hediste	Burrow_Volume_10_HD03	Burrow_Volume_10_HD03_1220_1235_854_	Burrow_Volume_10_HD03_1220_1235_854_000.tif	Burrow_Volume_10_HD03_1220_1235_854_853.tif
28	11	Rep_03	Hydrobia ulvae	1129	1188	332	332	Species_Hydrobia	Burrow_Volume_11_HU03	Burrow_Volume_11_HU03_1129_1188_332_	Burrow_Volume_11_HU03_1129_1188_332_000.tif	Burrow_Volume_11_HU03_1129_1188_332_331.tif
38	12	Rep_04	Mixed species	1228	1223	879	879	Species_Mixed	Burrow_Volume_12_Mx04	Burrow_Volume_12_Mx04_1228_1223_879_	Burrow_Volume_12_Mx04_1228_1223_879_000.tif	Burrow_Volume_12_Mx04_1228_1223_879_878.tif
22	13	Rep_04	Hediste diversicolor	1236	1222	943	943	Species_Hediste	Burrow_Volume_13_HD04	Burrow_Volume_13_HD04_1236_1222_943_	Burrow_Volume_13_HD04_1236_1222_943_000.tif	Burrow_Volume_13_HD04_1236_1222_943_942.tif
27	14	Rep_04	Hydrobia ulvae	1204	1151	305	305	Species_Hydrobia	Burrow_Volume_14_HU04	Burrow_Volume_14_HU04_1204_1151_305_	Burrow_Volume_14_HU04_1204_1151_305_000.tif	Burrow_Volume_14_HU04_1204_1151_305_304.tif
35	15	Rep_03	Corophium volutator	1204	1191	252	252	Species_Corophium	Burrow_Volume_15_CV03	Burrow_Volume_15_CV03_1204_1191_252_	Burrow_Volume_15_CV03_1204_1191_252_000.tif	Burrow_Volume_15_CV03_1204_1191_252_251.tif
32	16	Rep_04	Corophium volutator	1201	1168	274	274	Species_Corophium	Burrow_Volume_16_CV04	Burrow_Volume_16_CV04_1201_1168_274_	Burrow_Volume_16_CV04_1201_1168_274_000.tif	Burrow_Volume_16_CV04_1201_1168_274_273.tif
37	17	Rep_05	Mixed species	1228	1244	981	981	Species_Mixed	Burrow_Volume_17_Mx05	Burrow_Volume_17_Mx05_1228_1244_981_	Burrow_Volume_17_Mx05_1228_1244_981_000.tif	Burrow_Volume_17_Mx05_1228_1244_981_980.tif
33	18	Rep_05	Corophium volutator	1179	1195	332	332	Species_Corophium	Burrow_Volume_18_CV05	Burrow_Volume_18_CV05_1179_1195_332_	Burrow_Volume_18_CV05_1179_1195_332_000.tif	Burrow_Volume_18_CV05_1179_1195_332_331.tif
26	19	Rep_05	Hydrobia ulvae	1207	1140	335	335	Species_Hydrobia	Burrow_Volume_19_HU05	Burrow_Volume_19_HU05_1207_1140_335_	Burrow_Volume_19_HU05_1207_1140_335_000.tif	Burrow_Volume_19_HU05_1207_1140_335_334.tif
21	20	Rep_05	Hediste diversicolor	1235	1218	859	859	Species_Hediste	Burrow_Volume_20_HD05	Burrow_Volume_20_HD05_1235_1218_859_	Burrow_Volume_20_HD05_1235_1218_859_000.tif	Burrow_Volume_20_HD05_1235_1218_859_858.tif
